# Annual Meeting of the Ohio State Dental Society

**Published:** 1872-01

**Authors:** 


### Proceedings of Societies.

### ANNUAL MEETING OF THE OHIO STATE DENTAL SOCIETY.

The sixth annual meeting of the Ohio State Dental Society was held in the Temple of the I. O. O. F., at Columbus, December 5, 1871. The meeting was called to order at 11 o'clock A. M., by Dr. C. R. Taft, second Vice President, and proceedings were opened with prayer by Rev. Mr. Newton.

A large number responded to the call of the roll. The minutes of the preceding meeting were read and approved.

Dr. B. T. Spellman, chairman of Committee on Membership, requested all persons desiring membership to meet the committee after the morning session.

The Committee on Dental Ethics made a partial report, and were granted time.

On motion of Dr. J. Taft, a committee of three was appointed to take into consideration the advisability of holding a general quiz each day during the sessions of the body. The Chair appointed Drs. J. Taft, B. T. Spellman and E. C. Sloan.

On motion of Dr. Herriott, all dentists in the city were invited to sit as corresponding members.

Adjourned to meet at 2 o'clock P. M.

### AFTERNOON SESSION.

Meeting called to order by Dr. Jennings, first Vice President. Minutes read and approved.

On motion of Dr. Watt, the first subject for discussion was laid on the table, to be taken up at a future time, and the second subject, "Filling Teeth," was then taken up and discussed by Drs. Spellman, Watt and Herriott.

The Committee on "Quiz" reported as follows:

"Your committee would report that they have considered the subject of holding daily quizzes during the sessions of this meeting, and would recommend that a general exercise of this character be held twice each day, viz.: at the opening of the morning session and at the opening of the night session, to continue three-quarters of an hour each; the subject matter of such exercises being chiefly such as pertain to practice in its various details. They shall be conducted by persons appointed for the purpose. Your committee would suggest that the exercises be chiefly oral, but written questions are admissible. We would suggest as conductor for this evening's session, Dr. Geo. Watt; to-morrow morning, Dr. C. R. Butler; to-morrow evening, Dr. H. A. Smith; Thursday morning, F. H. Rehwinkle; Thursday evening, W. M. Herriott.

J. TAFT,
B. T. SPELLMAN,
E. C. SLOAN."

On motion, the report was adopted.

The discussion of "Filling Teeth" was continued by Drs. Smith, Rehwinkle, Blount, Buffett, C. R. Taft, N. W. Williams, I. Williams, Kelsey and Porter, after which C. R. Taft read an essay on Filling Teeth, which was well received by the meeting, and, on motion, was referred to the Committee on Publication. The essay was discussed by Drs. Blount, E. J. Waye, Watt, Butler, J. Taft and Spellman.

On motion of Dr. Blount, Dr. P. G. C. Hunt, of Indianapolis, Ind., was elected an honorary member of this Society.

The Committee on Membership reported the following as worthy of membership: Samuel Clippenger, of Toledo, and J. Frank McGinnis, of Bellefontaine. Report received and the persons duly elected by vote.

Adjourned to meet at 7½ o'clock P. M.

EVENING SESSION.

Minutes read and approved.

The evening "quiz," being the first thing in order, was conducted by Dr. Watt in a very spirited manner.

The subject under discussion at the close of the afternoon session was resumed and carried on by Drs. Butler, Watt, Herriott, Spellman, H. A. Smith, J. Taft and Clippenger.

On motion, the subject was laid over till to-morrow morning.

The Chair appointed Drs. J. Taft, C. R. Butler, A. A. Blount and P. G. C. Hunt Committee on Clinic Operations, to occupy the time from 8 A. M. till 10½ A. M. of the second day.

Adjourned to meet at 8 o'clock A. M. next day for clinics and 10½ A. M. for business.

MORNING SESSION—SECOND DAY.

Meeting called to order at 11 o'clock A. M. by Dr. Jennings. Minutes read and approved.

Dr. Spellman, from the Committee on Membership, reported the following as suitable for membership: J. W. Lyder, Alliance; Chas. Welch, Wilmington; J. H. Siddall, Canton; J. M. Segar, Akron; S. R. Beckwith, Kinsman, and E. L. Perry, Milan.

Report received and the persons duly elected.

Quiz was called by Dr. Butler, appointed to conduct it, who was, at his own request, excused.

Dr. J. Taft offered the following:

"*Resolved*, That two Normal-Class Clinical operators or teachers be appointed by the Society annually, whose duty it shall be to answer any calls by the profession in the State for clinical instruction, and any additional assistance practicable, whenever a sufficient number of the profession in any given locality shall unite in extending such invitation, and be responsible for the expense incurred in filling such requisition, including a salary for such services as shall hereafter be agreed upon."

After discussion by Drs. J. Taft, Butler, Watt, Rehwinkle, J. H. Warner, Berry and C. R. Taft, the resolution was adopted unanimously.

On motion, the Chair was instructed to appoint a committee of three to report upon suitable persons to act as clinical teachers for the coming year.

Adjourned to meet at 2 o'clock P. M.

AFTERNOON SESSION.

Minutes read and approved.

The Chair announced Drs B. T. Spellman, L. Buffett and C. R. Taft as committee on appointment of clinical teachers.

The certificate of Dr. J. G. Templeton, a member of the Pennsylvania State Dental Society, was presented by the Secretary, and, on motion of Dr. Watt, Dr. Templeton was invited to participate in the proceedings, and also to convey the kind greetings of this Society to the Pennsylvania State Dental Society.

Dr. Watt also moved the appointment of a delegate to the latter, and that Dr. Templeton be elected an honorary member of the Ohio State Dental Society. Adopted unanimously.

On motion, Dr. J. Taft was appointed delegate to the Pennsylvania State Dental Society in 1872.

On motion of Dr. Kelsey, the subject of "Filling Teeth" was passed, and the next subject, "The Treatment of Dental Irregularities," was taken up and fully discussed by Drs. Berry, Watt, Rosson, Herriott, J. H. Warner, Butler, Sedgwick, Buffett and Blount.

On motion, the subject was passed.

The Secretary read a letter from Dr. G. W. Keely, President, regretting his absence.

On motion of Dr. Blount, a committee consisting of Drs. D. R. Jennings, J. Taft and F. H. Rehwinkle, was appointed to draft resolutions expressive of the sense of this body relating to the departure of Dr. N. W. Williams, an active member of this Society, to a foreign field of professional usefulness.

On motion, Dr. Smith read a paper from Dr. Keely, President, which was received and referred to the Publishing Committee.

On motion of Dr. Spellman, Chairman Membership Committee, Dr. G. W. Cooly, of Athens, was recommended for membership, and, being balloted for, was elected.

On motion, a committee of three to nominate officers for the State Board of Examiners was appointed, as follows: B. F. Rosson, E. J. Waye and A. A. Blount.

On motion, a committee of five to nominate officers for State Society, consisting of M. DeCamp, W. P. Horton, Saml. Clippenger, H. A. Smith and N. W. Williams, was appointed.

On motion of Dr. Berry, the Rubber question was taken up, and discussed by Drs. Berry, Rehwinkle, Wright, J. H. Warner, H. A. Smith, Butler and Horton.

On motion of Dr. Horton, a committee of three was appointed to report to this Society what action it had better take upon the subject of rubber. The Chair appointed Drs. Horton, Rehwinkle and Berry.

Adjourned to meet at 7½ P. M.

### EVENING SESSION.

Society called to order by Dr. Jennings. Minutes read.

Dr. Smith being absent, on motion, Dr. Butler was called to conduct the quiz, which he did with interest and benefit to all present, as the subject of destroying nerves was pretty fully ventilated.

The committee to whom was referred the subject of Dr. Taft's resolution on clinical teachers reported, through Dr. Spellman, the names of Dr. Corydon Palmer, of Warren,

Dr. Will Taft, of Cincinnati, and Dr. C. R. Butler, of Cleveland.

On motion, the report was received and the persons named unanimously elected.

On motion of Dr. Watt, the subject "Dental Medicine" was called and discussed by Drs. Watt, Spellman, J. Taft and Butler.

On motion of Dr. DeCamp, the time of holding the election for State Society officers was changed until 1½ o'clock P. M. third day.

Adjourned to meet at 9 o'clock to-morrow.

### MORNING SESSION—THIRD DAY.

Society called to order by Dr. D. R. Jennings, President. Minutes read and approved.

The committee to select delegates to the American Dental Association reported the following persons, who were elected to represent this Society in said Association: I. Williams, A. Berry, S. Clippenger, B. F. Rosson, W. M. Herriott, E. J. Waye, E. C. Sloan, M. DeCamp, W. H. Sedgwick, F. H. Rehwinkle, A A. Blount, J. Taft, D. R. Jennings, A. F. Price, J. W. Lider. J. Buffett, W. H. Sedgwick, Committee.

On motion, the morning quiz was passed, and the subject, "Bases for Artificial Teeth," was taken up and discussed by Drs. Herriott, Watt, Kelsey, J. H. Warner, Clippenger and Rosson.

The hour having arrived to report officers for the coming year, M. DeCamp, Chairman, reported: The committee, B. F. Rosson, E. J. Waye and A. A. Blount, to report members of State Board of Examiners, report that they have fully considered the whole matter, and unanimously recommend that, in consideration of the excellent manner in which the present outgoing members, Dr. J. Taft and Dr. F. H. Rehwinkle, of this Board, have filled the office, they be elected, which, on motion, was unanimously concurred in by a rising vote.

Dr. Taft thanked the Society for this recognition of the efficiency of the Board.

On motion of Dr. Taft, it was resolved to pay a salary of $30 per annum to the Secretary of this Society for his services, this to include the Secretary of this present meeting.

The subject, "Dental Bases," was continued under discussion by Drs. Berry and J. Taft.

An essay was presented by Dr. H. A. Smith upon this subject.

Dr. Rosson gave a written notice that at the next annual meeting he would move the addition to the by-laws that officers of this Society shall be nominated in Committee of the Whole.

Dr. R. G. Warner, of Columbus, H. W. How, Chillicothe, and W. H. Carter, London, were recommended by the Committee on Membership, and unanimously elected.

Dr. Smith's paper was discussed by Drs. Herriott, Rehwinkle, Butler, Smith, Templeton, Watt.

N. W. Williams, C. R. Butler and B. F. Rosson were appointed an additional committee, to whom were referred all bills.

The committee appointed to give expression to the sense of this Society relative to the departure of Dr. N. W. Williams, would report:

"That in view of the fact that our esteemed professional brother, Dr. N. W. Williams, is about to change his field of labor from this to a foreign country, we are fully conscious that the profession of this State is about to lose a most valuable co-laborer in its earnest work of elevating and extending its usefulness. We are loth to part with him, and as colleagues, greatly regret his departure; but, as warm personal friends, wish him 'God speed,' and that he may realize all his hopes and expectations. We congratulate the community which may be fortunate enough to secure his professional services. His patients may rest assured that they are in the hands of a gentleman, who is in every respect qualified to represent American Dentistry anywhere.

"D. R. Jennings,
"F. H. Rehwinkle,
"J. Taft."

Adjourned to meet at 1½ o'clock P. M.

### AFTERNOON SESSION.

Minutes read and approved.

Dr. N. W. Williams, from the Auditing Committee, reported bills to the amount of $133.50, which were ordered to be paid.

The hour having arrived for the election of officers, balloting was had, with the following result:

B. F. Spellman, Warren, President.

I. Williams, New Philadelphia, 1st Vice President.

E. C. Sloan, Ironton, 2d Vice President.

C. R. Taft, Mansfield, Recording Secretary.

C. R. Butler, Cleveland, Corresponding Secretary.

L. Buffett, Cleveland, Treasurer.

On motion, Dr. Watt was called to conduct the President-elect to the chair.

The Committee on Rubber reported the following:

"Your Committee on the Rubber matter would report that, in their opinion, it is abvisable for the Society to choose a committee of five to have full control and take such measures in reference to resisting the claims of the Rubber Company, and employ a lawyer, and to take steps for obtaining funds, &c., as the committee may deem proper, and report at the next meeting of the Society.

W. P. Horton,

A. Berry,

F. H. Rehwinkle, } *Committee.*

On motion, the report was accepted and approved, and F. H. Rehwinkle, H. A. Smith, W. P. Horton, I. Williams and G. W. Keely were appointed said committee.

The committee appointed to take action on the death of Dr. Dunn made the following report, which, on motion, was adopted:

"Whereas, It has pleased Almighty God, in whose hands are all things and whose ways are past finding out, to remove from his earthly labors Dr. G. W. Dunn, a member in good standing of this Society; therefore be it

"*Resolved*, That the Ohio State Dental Society hears with deep regret of his departure 'beyond that bourne from whence no traveler returns.'

"*Resolved*, That the heartfelt sympathy of this Society is hereby tendered the members of the bereaved family in their great loss.

"*Resolved*, That this preamble and resolutions be engrossed upon the minutes of this Society, and that the Secretary is hereby requested to forward a copy of the same to the family of the deceased.

On motion, an appropriation of one hundred and fifty dollars was placed at the disposal of the Committee on Rubber.

On motion of Dr. Watt, Dr. C. H. James was elected an honorary member of this Society.

The Chair announced the following standing committees for the ensuing year:

Executive Committee—L. Buffett, B. F. Rosson, R. G Warner.

On Membership—I. Williams, A. A. Blount, J. M. Porter.

On Publishing and Dental Ethics—J. Taft, Geo. Watt, C. R. Butler.

Dr. Watt, the retiring Treasurer, reported as follows: Balance on hand after paying all bills allowed $241.17; transferred to Treasurer elect, $241.17.

On motion, Drs. Will. Taft, M. DeCamp and C. Palmer were appointed a committee to obtain the names of all dentists in the State.

The Board of Examiners made the following reports, which, on motion, were accepted:

The Examining Board begs leave to report that it has held two meetings since the annual meeting of December 7th and 8th, 1870. The first was at Cleveland, on the first Wednesday, being the 7th day of June, 1871, at which the following named candidates appeared for examination, viz.: J. E. Robinson, Rollin Horton, S. B. Burnham, R. R. Peebles, Cleveland, Cuyahoga county; Dow F. Wemple, Sandusky, Erie county; J. T. Barclay, Robert Bell, Columbiana, Columbiana county; C. B. Knowlton, Elyria, Lorain county; S. B. Beckwith, Kinsman, Trumbull county; H. S. Clark, Beverly, Washington county; James H. Peterson, S. D. Stewart, Akron, Summit county; Charles R. Sabin, Lima, Allen county; Wm. Beilharz, Bucyrus, Crawford county; E. Tibbitts, Cincinnati, Hamilton county; D. T. D. Dyshe, Lebanon, Warren county. These, after full examination, were granted certificates to practice dentistry in this State.

The name of W. A. Porter, Dalton, Wayne county, was inadvertently omitted from the list of those examined at the annual meeting, and is here inserted.

The second meeting was held at the American House, Columbus, on the 6th and 7th days of December, 1871, at which there appeared for examination the following named candidates, viz.: George W. Cooley, Athens, Athens county; J. M. Segur, Akron, Summit county; E. L. Perry, Milan,

Erie county; H. W. Howe, Chillicothe, Ross county; Chas. C. Beilharz, Tiffin, Seneca county; Wm. H. Carter, London, Madison county; C. H. Leaman, Dayton, Montgomery county; R. G. Warner, Columbus, Franklin county; Sam'l T. Boggess, Jackson, Jackson county; L. T .Beilharz, Fremont, Sandusky county; James J. Erwin, Newton Falls, Trumbull county; Fredrick K. Thorpe, Cleveland, Cuyahoga county; George Hall, Lima, Allen county; L. D. Converse, Urbana, Champaign county; Jesse A. Dunn, Columbus, Franklin county; W. W. Woodward, Cincinnati, Hamilton county.

These, after due examination, were granted certificates to practice dentistry.

Respectfully submitted,

J. Taft, President.

W. P. Horton, Secretary.

December 7th, 1871.—The Board of Examiners would submit the following financial report as a summing up of its whole transactions to date:

| | |
|---|---|
| Number of Certificates issued, 56 at $25 . . | $1,400 00 |
| Expenses for printing, stationery, stamps, and per diem for members of Board, . . . | 922 45 |
| Paid cash Treasurer of State Society, . . | 477 55 |
| | $1,400 00 |

Respectfully submitted,

J. Taft, President.

W. P. Horton, Treasurer.

On motion, the Society then adjourned to meet on the first Wednesday in December, 1872.

